# Silencing of the *XAF1 *gene by promoter hypermethylation in cancer cells and reactivation to TRAIL-sensitization by IFN-β

**DOI:** 10.1186/1471-2407-7-52

**Published:** 2007-03-21

**Authors:** O Cristina Micali, Herman H Cheung, Stéphanie Plenchette, Sandra L Hurley, Peter Liston, Eric C LaCasse, Robert G Korneluk

**Affiliations:** 1 Department of Plant-Microbe Interactions, Max-Planck Institute for Plant Breeding Research, Koeln, Germany; 2 Apoptosis Research Centre (ARC), Children's Hospital of Eastern Ontario Research Institute, Ottawa, Canada

## Abstract

**Background:**

XIAP-associated factor 1 (XAF1) is a putative tumor suppressor that exerts its proapoptotic effects through both caspase-dependent and – independent means. Loss of XAF1 expression through promoter methylation has been implicated in the process of tumorigenesis in a variety of cancers. In this report, we investigated the role of basal *xaf1 *promoter methylation in *xaf1 *expression and assessed the responsiveness of cancer cell lines to XAF1 induction by IFN-β.

**Methods:**

We used the conventional bisulfite DNA modification and sequencing method to determine the methylation status in the CpG sites of *xaf1 *promoter in glioblastoma (SF539, SF295), neuroblastoma (SK-N-AS) and cervical carcinoma (HeLa) cells. We analysed the status and incidence of basal *xaf1 *promoter methylation in *xaf1 *expression in non-treated cells as well as under a short or long exposure to IFN-β. Stable XAF1 glioblastoma knock-down cell lines were established to characterize the direct implication of XAF1 in IFN-β-mediated sensitization to TRAIL-induced cell death.

**Results:**

We found a strong variability in *xaf1 *promoter methylation profile and responsiveness to IFN-β across the four cancer cell lines studied. At the basal level, aberrant promoter methylation was linked to *xaf1 *gene silencing. After a short exposure, the IFN-β-mediated reactivation of *xaf1 *gene expression was related to the degree of basal promoter methylation. However, in spite of continued promoter hypermethylation, we find that IFN-β induced a transient *xaf1 *expression, that in turn, was followed by promoter demethylation upon a prolonged exposure. Importantly, we demonstrated for the first time that IFN-β-mediated reactivation of endogenous XAF1 plays a critical role in TRAIL-induced cell death since XAF1 knock-down cell lines completely lost their IFN-β-mediated TRAIL sensitivity.

**Conclusion:**

Together, these results suggest that promoter demethylation is not the sole factor determining *xaf1 *gene induction under IFN-β treatment. Furthermore, our study provides evidence that XAF1 is a crucial interferon-stimulated gene (ISG) mediator of IFN-induced sensitization to TRAIL in cancer.

## Background

Cell life and death decisions rely on a delicate balance between pro- and anti-apoptotic factors. A disruption of this balance can result in a variety of pathologies including cancer, autoimmune and neurodegenerative diseases. In cancer cells, anti-apoptotic factors such as the Inhibitors of Apoptosis (IAPs) render cells resistant to apoptosis, primarily through their inhibition of core death executioners, the caspases, or through the neutralization of antagonists such as Smac/DIABLO and Omi/HtrA2 [1]. In particular, Baculovirus IAP Repeat domains (BIRs) present in XIAP can interact directly and inhibit initiator and/or effector caspases. In addition, the RING finger domain present in some IAPs such as XIAP and cIAP-1 acts as an E3 ubiquitin ligase, targeting the IAP-caspase complex for degradation via the proteasome [2]. Thus, IAP function is central to the modulation of the apoptotic cascade.

To counter the effects of the IAPs, several antagonists such as XIAP-Associated Factor-1 (XAF1), Smac/DIABLO and Omi/HtrA2 have been identified that play essential roles in apoptosis [3]. XAF1 has been independently identified in several screens as a key mediator of apoptosis [4-6] and is shown to dramatically sensitize cancer cells to apoptotic triggers such as TNF-related apoptosis-inducing ligand (TRAIL) and etoposide treatments [4,7]. This sensitization is in part achieved through XAF1 inhibition of XIAP anti-caspase activity. In addition, XAF1 also appears to enhance the apoptotic effects of TNF-α independently of its interaction with XIAP [8]. Furthermore, in urogenital cancers, XAF1 was recently shown to quicken the apoptosis response through its enhancement of p53 protein stability [6]. These recent findings identify XAF1 as a candidate tumor suppressor at the junction of several major pathways leading to apoptosis.

The loss of XAF1 is associated with malignant tumor progression in a variety of cancers. XAF1 levels are drastically decreased in a significant number of cancer cell lines [5,9], as well as in a collection of gastric cancers [10], melanoma specimens [5] and urogenital cancers [6]. Loss of XAF1 is due, at least in part, to epigenetic alterations such as DNA methylation at several CpG sites within the promoter region [6,10]. In gastric cancers, the relative decrease in *xaf1 *transcript level correlates with the stage and grade of the tumor, suggesting that loss of XAF1 contributes to the process of tumorigenesis [10]. It is therefore predicted that methods that enhance XAF1 levels could increase apoptotic susceptibility and provide an additional strategy for cancer therapy.

In this context, the discovery of *xaf1 *as an Interferon Stimulated Gene (ISG) provides evidence for the feasibility of such a therapeutic strategy. The induction of XAF1 by IFN-β in human melanoma cell lines results in enhanced susceptibility to TRAIL-induced apoptosis [7]. Expression of a truncated XAF1 protein lacking part of a zinc-finger domain abrogates IFN-dependent sensitization to TRAIL, suggesting that XAF1 plays an essential role in IFN-mediated apoptosis. A potential pitfall of the use of IFN to induce *xaf1 *expression is the frequent hypermethylation of the *xaf1 *promoter observed in many cancer cell lines [6,10]. Promoter hypermethylation can lead to transcriptional gene silencing through the assembly of condensed chromatin domains or through simple inhibition of transcription factor binding [11]. DNA methylation and the subsequent silencing of several IFN pathway components including RNAseL and RNA-activated protein kinase (PKR) has been identified as a major mechanism associated with carcinogenesis [12,13]. The reversal of the DNA methylation status, through treatment with the methyltransferase inhibitor 5-aza-2'-deoxycytidine (5-AZA-dC) can restore cell susceptibility to IFN [13,14]. Although in the large majority of cases there is a strong correlation between promoter methylation and resistance to induction by IFN, exceptions exist. In neuroblastoma cells, treatment with IFN-γ can induce methylation-silenced caspase-8 expression without affecting the methylation status within the promoter region [15]. As a plausible explanation, an alternative IFN-inducible promoter element has been found upstream of the first exon of *caspase-8 *gene and has been shown to be responsive to IFN-γ, independently of the methylation status [15,16].

In this report, we investigate the effect of IFN-β on the methylation status of *xaf1 *promoter and on the expression of *xaf1 *in SF539 and SF295 glioblastoma, SK-N-AS neuroblastoma and HeLa cervical carcinoma. In cancer cells, we find a strong correlation between methylation status of the *xaf1 *promoter and baseline gene expression at the transcript level. HeLa and SK-N-AS cell lines show the highest basal promoter hypermethylation among the four cancer cell lines studied compared to control cells, and are used as cellular models for studying the responsiveness of IFN-β-mediated XAF1 induction. In spite of basal promoter hypermethylation, IFN-β could induce *xaf1 *expression in HeLa and SK-N-AS. Futhermore, we demonstrate that IFN-β induced a transient increase in the *xaf1 *transcript and protein levels apparent at 48 hours post-treatment. However, we do not find a tight link between IFN-β-mediated *xaf1 *gene induction and decrease in average methylation within *xaf1 *promoter region in HeLa and SK-N-AS cells occurring at later time points. These results suggest that *xaf1 *expression would be positively regulated by additional mechanisms other than promoter demethylation. To further understand the importance of *xaf1 *gene induction in IFN-β-sensitization to TRAIL-mediated cell death, we established stable glioblastoma SF539 cell lines carrying specific *xaf1 *shRNA. We find that *xaf1 *knockout stable SF539 clones are unresponsive to IFN-β-mediated XAF1 induction, rendering these stable cell lines resistant to sensitization by IFN-β to TRAIL-mediated cell death. Our study clearly demonstrates that *xaf1 *is a critical ISG implicated in IFN-β-mediated sensitization to TRAIL-induced cell death.

## Methods

### Cell culture and transfection

Glioblastoma (SF539 and SF295) and neuroblastoma (SK-N-AS) cell lines were maintained on Dulbecco's Modified Eagle Medium (DMEM), 10% foetal bovine serum (FBS), supplemented with 4 mM glutamine and 0.1 mM non-essential amino-acids. Cervical carcinoma (HeLa) cells were maintained in Minimal Essential Medium (MEM), 10% FBS supplemented with 2 mM glutamine and 0.1 mM non-essential amino acids. For transfection, cells were grown to 70%–80% confluency in 6-well plates and transfected with linearized plasmid DNA using Lipofectamine 2000 (Invitrogen, Burlington ON, Canada) according to the manufacturer's recommendations. Stable clones were selected in DMEM supplemented with 600 μg/mL G418 (Invitrogen).

When required, IFN-β (R&D Systems, Minneapolis, MN, USA) was added at a concentration of 500 U/mL and 5-aza-2'-deoxycytidine (Sigma-Aldrich) at the doses indicated.

### Xaf1 short hairpin RNA (shRNA) plasmid construction and screening

The design and construction of the shRNA clones against *xaf1 *was performed according to the shagging-PCR method described previously [17]. PCR amplification reactions were performed on U6 promoter template using a forward primer (5'-AGGAGATCTGCGCAGGCAAAACGCACCAC-3') and a variety of 96 nucleotide-long shRNA *xaf1 *primers based on a similar approach described previously for shRNA XIAP [18]. PCR products were cloned into a TOPO-TA vector (Invitrogen) and were screened for activity by transient transfection into 293T cells followed by *xaf1 *transcript quantification by quantitative RT-PCR. One of the constructs that caused a significant decrease in *xaf1 *transcript levels was selected for stable transfection into glioblastoma SF539 cells. The construct targets exon-2 that is shared among the three major splice isoforms of *xaf1 *(5'-CCATGGAGGAGCACTGCAAGCTTGAGCAC-3'). The shRNA was sub-cloned into a pCDNA3 vector devoid of its CMV promoter [18] and transfected into SF539 cells as described above.

### Quantitative RT-PCR

Quantitative RT-PCR using *xaf1*-specific primers and the TaqMan method [19] was performed as described previously [9]. Briefly, total RNA was extracted from 1 × 10^7 ^cells using the RNeasy kit (Qiagen, Mississauga ON, Canada). 100 ng aliquots were assayed by reverse-transcription followed by PCR (TaqMan EZ-RT PCR kit, Applied Biosystems, Foster City CA, USA) with *xaf1*-specific primer/probe combinations [9]. GAPDH primer and probes were used as a control. All assays were performed at least in triplicate.

### Western blot analysis

Cells were grown in 6-well plates at a density of ~7 × 10^5 ^cells per well. Cells were either treated or not with IFN-β (500 U/mL) for 24 hours or more, harvested by scraping, washed twice in PBS (pH 7.2) and lysed with boiling lysis buffer (1% SDS, 1 mM ortho-vanadate, 10 mM tris pH 7.4). Protein concentration was determined with the RC DC Bradford assay (BioRad, Montreal QC, Canada). 20 μg to 30 μg of total protein in SDS sample buffer was resolved by SDS-PAGE (10% or 12.5%) and transferred to PVDF membrane using a semi-dry transfer system (Hoepher SemiPhor). Membranes were blocked overnight in PBS (0.1% Tween, 5% skim milk) and were incubated with primary antibody (PBS, 2% skim milk) for 12 to 16 hours at 4°C. The membrane was incubated with secondary antibody (PBS, 2% skim milk) for two hours and the signal was revealed by enhanced chemiluminescence (ECL) or ECL-Plus kits (Amersham Biosciences, Baie d'Urfe, QC, Canada). Mouse monoclonal anti-XAF1 and rabbit polyclonal anti-XIAP antibodies were generated against their respective full-length GST-tagged construct following standard techniques. Antibodies against β-actin and GAPDH were obtained from Sigma and Advanced Immunochemical, respectively.

### Bisulphite DNA sequencing

Cells were grown to 80% confluency in 10 cm plates and harvested by scraping. Genomic DNA was extracted using the DNeasy kit (Qiagen). Approximately 1 μg of DNA was modified with sodium bisulphite as outlined previously [20]. Modified DNA was used as a template in PCR reactions with primers MS2 and MS3 [10]. PCR products were cloned into TOPO-TA vector and transformed into *E. coli *XL-10 gold cells (Stratagene) and 10 positive clones were randomly selected for sequencing. Methylation status was assessed at eight CpG sites situated within a 250 bp region upstream of the translational start site, whose methylation status was previously shown to correlate with *xaf1 *gene silencing [10]. As a control, DNA was extracted from buffy coat preparations of blood samples donated by two healthy volunteers. Control DNA was processed as described above.

### Cell viability assay

Cells were seeded in 96 well plates at 1 × 10^5 ^cells per well (100 μl of medium) and were allowed to adhere for at least 8 hours. Growth medium was replaced with medium containing TRAIL at concentrations ranging from 0 to 100 ng/mL and cell viability was assessed 16 to 20 hours later by the WST-1 assay (Roche Diagnostics, Laval QC Canada).

## Results

### Relationship between basal profile of *xaf1 *promoter methylation and IFN-β-mediated induction of *xaf1 *expression

**Figure 1:**
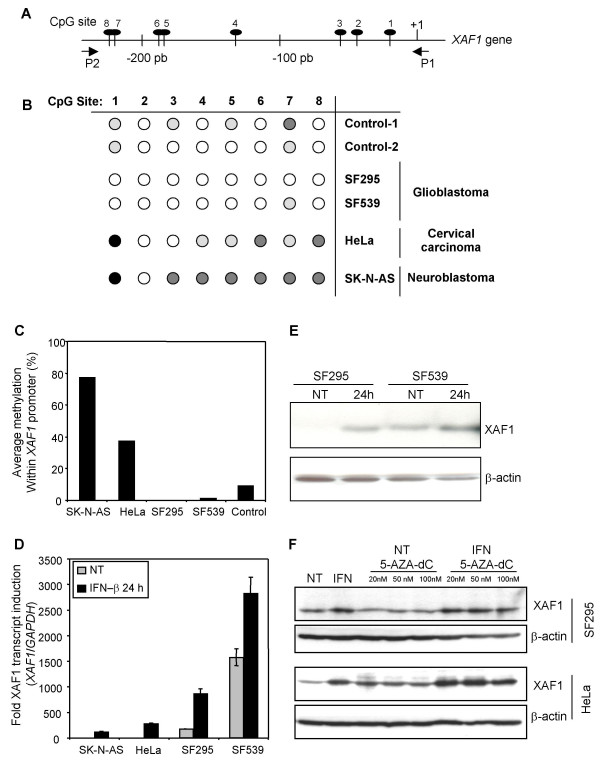
**Effect of IFN-β treatment on *xaf1 *transcript and protein levels in selected cell lines**. (A) Schematic representation of the 8 CpG sites of the 5' upstream region of the *xaf1 *gene. (B) Methylation status of *xaf1 *promoter region in selected cell lines was determined based on analysis of 8 CpG sites by methylation-sensitive PCR. Genomic DNA was treated with sodium bisulphite. PCR products with MS2 (P1) and MS3 (P2) primers were cloned into the TopoTA vector and sequenced. Ten cloned PCR products were sequenced for each cell line. Black, dark gray/light gray and open circles represent complete methylation, partial methylation and unmethylation at each CpG site, respectively. Controls are total blood DNA from healthy volunteers. (C) Graph representing the percentage of average methylation at all sites in selected cell lines. (D) *xaf1 *transcript levels were assessed by quantitative RT-PCR on total RNA and indexed to GAPDH transcript levels in non-treated cells (NT) and cells exposed to 500 U/mL of IFN-β for 24 hours. The protein level of endogenous (E) or induced (F) XAF1 was determined by western blot analysis, 30 μg of total protein extract were resolved by 12% SDS-PAGE and XAF1 was detected using an anti-XAF1 mouse monoclonal antibody. Membranes were stripped and re-probed with anti-β-actin antibody as a loading control. (F) Cells were left untreated (NT), exposed to 500 U/mL of IFN-β (IFN) for 24 hours or treated with 5-AZA-dC at the indicated concentration, followed 24 hours later by IFN-β for 24 hours. The blot in (E) was intentionally overexposed to increase the weak signal of XAF1 in SF295 cells (F).

Four cancer cell lines, SF295, SF539, SK-N-AS and HeLa were selected to investigate the effect of *xaf1 *promoter methylation on IFN-β mediated *xaf1 *induction. Of these, glioblastoma SF295 cells have previously been shown to carry little or no detectable XAF1 protein [4]. In contrast, the SF539 cell line expresses substantial amounts of XAF1 protein, compared to other cancer cell lines examined [4]. In order to assess the degree of *xaf1 *promoter methylation within these cell lines, a bisulphite DNA modification and sequencing was performed. The methylation status of 8 CpG sites situated within a 250 bp region immediately upstream of the translational start and corresponding to part of the *xaf1 *promoter was PCR amplified and 10 PCR clones were isolated and sequenced (Figure 1A and 1B). The methylation status was compared to control total blood DNA obtained from healthy volunteers. Cancer cell lines tested vary extensively in their degree of methylation (Figure 1B). HeLa (average methylation at all sites 37.5%) and SK-N-AS (77.5%) cells display significantly more methylated CpG sites than control samples (9.3%), SF295 (0%) and SF539 (1.3%) (Figure 1C). The degree of promoter methylation is inversely correlated with *xaf1 *transcript levels measured by quantitative RT-PCR (Figure 1D). Cell lines SF539 and SF295 that display low promoter methylation have comparatively more *xaf1 *transcript than HeLa and SK-N-AS cell lines.

To assess the effect of IFN-β on *xaf1 *gene expression in the context of basal *xaf1 *promoter methylation, cells were treated with 500 U/mL IFN-β for 24 hours and *xaf1 *transcript levels were measured by quantitative RT-PCR (Figure 1D). A strong inverse correlation exists between basal *xaf1 *promoter methylation and transcription induction by IFN-β. Cell lines displaying high levels of basal methylation (e.g. SK-N-AS and HeLa) show minimal *xaf1 *induction, whereas cell lines with no or little methylation display enhanced levels of *xaf1 *transcript (e.g. SF295 and SF539) in response to IFN-β. IFN-β treatment also causes a significant increase in XAF1 protein level in cell lines displaying low levels of basal promoter methylation (Figure 1E). XAF1 protein induction is not detectable in cell lines with very high levels of promoter methylation (data not shown).

To assess the effect of IFN-β on XAF1 protein expression under demethylating conditions, we have compared the effects of the DNA methylation inhibitor 5-AZA-dC and IFN-β treatment alone and in combination. Treatment with 5-AZA-dC alone up-regulated the expression of XAF1 in HeLa cells (hypermethylated *xaf1 *promoter at baseline) but had no effect in SF295 (hypomethylated *xaf1 *promoter at baseline) used as a control. The combination of 5-AZA-dC and IFN-β synergistically induced XAF1 expression in HeLa cells. However, no such effect was detected in cell lines lacking *xaf1 *promoter methylation (SF295) (Figure 1F). Treatment with IFN-β induced XAF1 expression regardless of the promoter methylation status of the cells suggesting a more complex mechanism of *xaf1 *transcriptional regulation.

### Methylation dynamics of xaf1 promoter in cancer cell lines after prolonged IFN-β treatment

**Figure 2:**
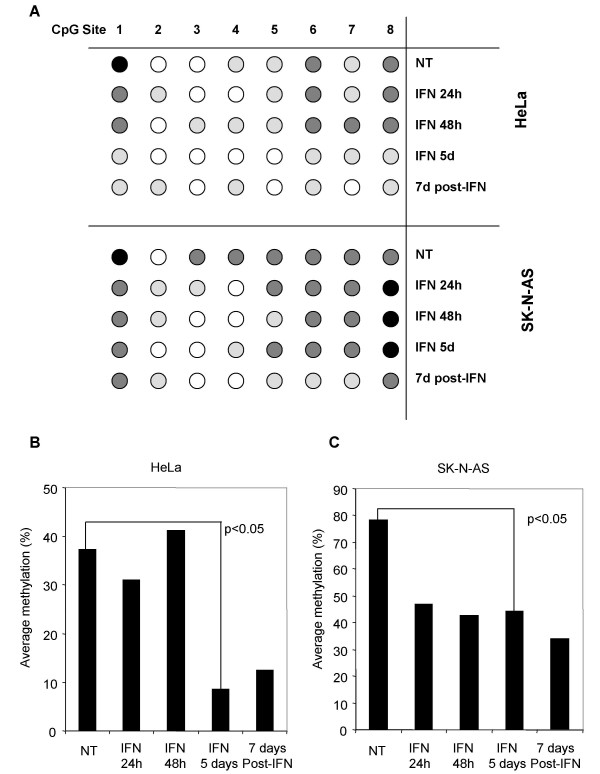
**Effect of IFN-β on methylation dynamics of *xaf1 *promoter**. Cells were exposed to 500 U/mL of IFN-β in culture medium for 0 hour, 24 hours, 48 hours or 5 days. (A) The methylation status of *xaf1 *promoter region in HeLa and SK-N-AS cells was determined based on the analysis of 8 CpG sites, by methylation-sensitive PCR as described previously in Figure 1. (B) HeLa and (C) SK-N-AS cells were treated for 5 days, and then were subsequently sub-cultured on medium lacking IFN-β for 7 days. Genomic DNA was harvested and subjected to sodium bisulphite treatment and PCR using primers MS2 and MS3. Results represent average methylation for 10 independent clones at 8 representative CpG sites for each cell line. After 5 days of continuous exposure to IFN-β there is a significant overall drop (p < 0.05) in methylation within the *xaf1 *promoter region.

The observation that cell lines heavily methylated within the *xaf1 *promoter still respond to IFN-β treatment (i.e. 72- and 266-fold *xaf1 *transcript induction in SK-N-AS and HeLa cells, respectively, Figure 1C) suggested that transcriptional silencing associated with *xaf1 *promoter methylation may be reversible. To address this possibility, we surveyed *xaf1 *promoter methylation status in HeLa and SK-N-AS cells exposed of 500 U/mL IFN-β for up to five days, followed by a seven-day recovery in medium without IFN-β. A continuous five day treatment with IFN-β caused a significant demethylation of most previously methylated CpG sites that was confirmed by bisulfite DNA modification and sequencing analysis (Figure 2A). Indeed, IFN-β caused a net 76% and 44% reduction in promoter methylation in HeLa and SK-N-AS cells, respectively (Figures 2A and 2B). After 7 days without IFN-β, the level of methylation remained unchanged, indicating that the demethylated status of *xaf1 *promoter was stable.

**Figure 3:**
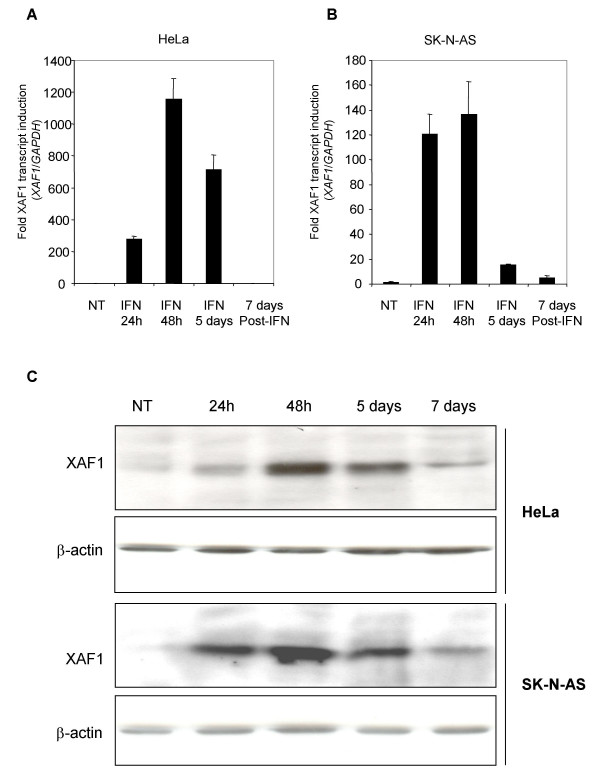
**Effect of IFN-β exposure on *xaf1 *expression at the transcript and protein levels**. HeLa (A) and SK-N-AS (B) cells treated with 500 U/mL IFN-β for 0 hour, 24 hours, 48 hours or 5 days, or after a 7-day IFN-β free period were harvested, total RNA was extracted and subjected to quantitative RT-PCR and indexed to GAPDH transcript levels. (C) 30 μg of total protein extract were resolved by 12% SDS-PAGE and XAF1 was detected using an anti-XAF1 mouse monoclonal antibody. Loading was checked by using an anti-β-actin monoclonal antibody.

Next, we analyzed the expression levels of *xaf1 *transcript (Figures 3A and 3B) and protein (Figure 3C) following the 5-day IFN-β treatment and 7-day recovery. Intriguingly, in the presence of IFN-β, there is no discernible correlation between promoter demethylation and transcript increase (Figures 2 and 3). *Xaf1 *transcript and protein levels are correlated to each other, reaching a peak at 48 hours and declining subsequently in both cell lines. Moreover, after IFN-β removal and despite sustained promoter demethylation, *xaf1 *transcript and protein levels continue to decrease. Thus, although IFN-β treatment resulted in a sustained loss of DNA methylation within the promoter region of *xaf1*, this treatment was not sufficient in maintaining increased *xaf1 *transcript or protein levels in the long term.

### Endogenous XAF1 is critical for IFN-β-mediated TRAIL-dependent apoptosis

**Figure 4:**
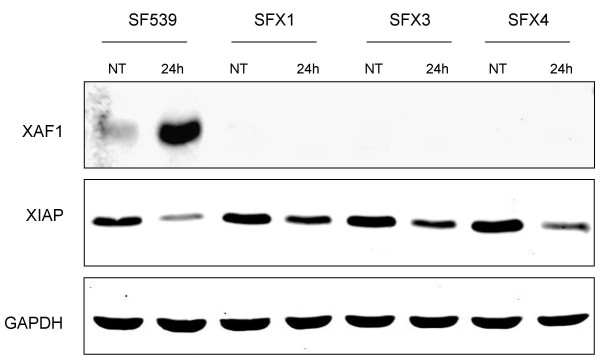
**Stable XAF1 knockout cell lines are resistant to IFN-β-mediated XAF1 induction**. The parental SF539 cell line and 3 stable *xaf1 *shRNA clones (SFX1, SFX3, SFX4) were exposed to 0 or 500 U/mL IFN-β for 24 hours. Total protein extracts were resolved by 12% SDS-PAGE and sequentially probed with XAF1, XIAP and GAPDH antibodies.

To place *xaf1 *in a therapeutic context, we evaluated the role of *xaf1 *expression in susceptibility to TRAIL-induced apoptosis in glioblastoma cells SF539. A previous study has shown that overexpression of *xaf1 *promotes TRAIL-induced apoptosis in melanoma cells [7]. Here, glioblastoma cells SF539 that display relatively high levels of *xaf1 *transcript, XAF1 protein and very low levels of promoter methylation were assessed for their susceptibility to TRAIL-induced apoptosis before and after shRNA-mediated *xaf1 *silencing. Due to the poor efficiency of transfection (20 to 30%) of the SF539 cells, stable clones (SFX1, SFX3, SFX4) expressing a shRNA construct directed against *xaf1 *were generated. These clones display a complete downregulation of endogenous XAF1 protein levels and abrogate XAF1 induction even after 24 hours of IFN-β treatment (Figure 4). Interestingly, treatment with IFN-β also causes a significant decrease in XIAP protein levels that appears to be at least in part dependent on the induction of XAF1, since this decrease is less marked in shRNA stable cell lines lacking XAF1.

**Figure 5:**
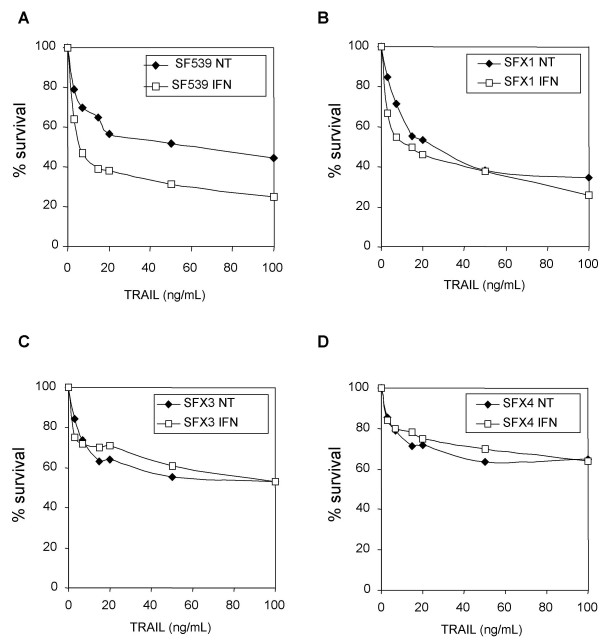
**XAF1 is essential for IFN-β-mediated TRAIL-dependent apoptosis**. SF539 (A) and 3 stable *xaf1 *shRNA clones (B-D) were treated with 0 or 500 U/mL of IFN-β for 24 hours prior to exposure to TRAIL for 14 hours. Cell viability was measured with the WST-1 assay as described in the methods and materials section. All assays were performed in triplicate and experiments were repeated at least three times.

Three of the stable *xaf1 *shRNA clones as well as the parental SF539 cell line were subjected to various concentrations of TRAIL with or without prior IFN-β treatment. As expected, IFN-β sensitized SF539 cells to TRAIL-induced cell death throughout the range of concentrations used in this experiment (Figure 5). In contrast, all three stable cell lines display a loss of IFN-β-mediated sensitivity to TRAIL, even though they differ in their TRAIL resistance in the absence of IFN-β. Together, these results demonstrate that the induction of endogenous XAF1 by IFN-β plays a critical role in sensitizing cancer cells to TRAIL-induced cell death.

## Discussion

In this study we have explored the relationship of XAF1 expression to gene silencing, promoter methylation and gene induction by IFN-β. Previous studies had indicated that loss of *xaf1 *expression, due at least in part to promoter methylation, is associated with increased tumor severity in gastric [10] and urogenital cancers [6]. In addition, XAF1 was shown to play a key role in the mechanism of IFN-β-associated sensitization to apoptotic cell death induced by TRAIL [7]. More recently, XAF1 was demonstrated to be critical in overcoming resistance to apoptosis induction by IFN-β in renal carcinoma and melanoma cells [21].

First, we validated the relationship between *xaf1 *silencing and promoter hypermethylation across four cancer cell lines HeLa, SK-N-AS, SF539 and SF295, that was to date, unexplored in this context. Then, we demonstrated that reverse methylation is not an absolute prerequisite for IFN-β-mediated XAF1 induction, since we showed a transient induction of XAF1 at both transcript and protein levels in spite of aberrant methylation, more particularly in HeLa cells. Finally, our results strengthen the importance of XAF1 expression in IFN-β-mediated sensitization to TRAIL-induced cell death in cancer cells.

Our current analysis of the methylation status of the *xaf1 *promoter is limited to the examination of 8 CpG sites within the proximal promoter region. These CpG sites have been previously validated to be more tightly associated with *xaf1 *expression compared with others distal CpG sites investigated [10]. In addition, very recently, 2 of the 8 CpGs at -2^nd^, -1^st ^positions (with respect of the transcriptional start site) were found to be more important in *xaf1 *transcriptional regulation [22]. However, further studies combining site-directed mutagenesis with methylation techniques will be necessary to ascertain the relevance of these sites in order to demonstrate if some of them are functionally more important than others.

Our results indicate that mechanisms, in addition to DNA methylation, are responsible for the control of XAF1 expression in cancer cells. Consistent with the pleitropic nature of IFN-β, multiple signalling pathways can be expected to be activated upon IFN-β challenge. For example, a large body of evidence has shown that the interaction of IFN-β with its receptor complex induces the transcription of ISGs through a signalling pathway involving the activation of the Janus kinase (JAK) family that in turn, phosphorylate substrate proteins called STATs (signal transducers and activators of transcription) [23]. In addition, the family of transcription factors known as interferon regulatory factors (IRFs) contribute to the numerous biological functions of IFN-α and IFN-β, which then modulate different sets of genes including *IFN-α*/*β *and many ISGs genes [24,25]. Interestingly, after treatment of human hepatoma cells with a combination of IFN-α and IFN-γ, transcriptional induction of selective ISGs (including *xaf1*) was found to be dependent upon IRF-1 [26].

In accordance with previous reports, we find a strong inverse correlation between *xaf1 *promoter methylation and baseline transcript in four cancer cell lines tested. In spite of the high level of promoter methylation in HeLa and SK-N-AS cell lines, a significant increase in *xaf1 *transcript and protein levels was detected following IFN-β treatment. Although the responsiveness to IFN-β-mediated XAF1 induction is generaly more effective in cells associated with low basal *xaf1 *promoter methylation, our results indicate that reverse methylation is not the sole factor modulating XAF1 expression. In this regard, we demonstrate that IFN-β has the ability to overcome the epigenetic modification of hypermethylation. Consistent with this result, IFN was previously found to mediate the induction of caspase-8 in neuroblastomas, without affecting promoter methylation [15,16]. Nevertheless, prolonged exposure to IFN-β leads to significant and stable demethylation of the *xaf1 *promoter, but long-term expression of the *xaf1 *transcript and protein remain unaffected by the treatment. We have attempted to provide explanation as to the mechanisms that governs *xaf1 *demethylation under IFN-β treatment, with respect to the role of DNMTs known in maintaining genes like *xaf1 *silenced [21,27]. We have demonstrated a synergistic effect of treatments combining the inhibitor of DNMTs, 5-AZA-dC, and IFN-β on XAF1 re-expression in cells with hypermethylated *xaf1 *promoter. This suggests that IFN-β may overcome DNMT activity to a certain extent, a finding that is consistent with a previous study showing that selective depletion of DNMT1 leads to demethylation and subsequent reactivation of *xaf1 *expression. Moreover, in this context, IFN-β-induced apoptosis was found to be dependent on *xaf1 *expression [21]. *Xaf1 *promoter methylation represents an initial mechanism of apoptosis resistance displayed by cancer cells. In this regard, it would be interesting to examine the correlation between DNA methyltransferases expression and *xaf1 *promoter methylation status in various cancer cell lines to evaluate their response to IFN-β-induced apoptosis.

These findings stress the importance of factors independent from DNA methylation that directly or indirectly control *xaf1 *expression. Very recently, studies have reported new transcriptional regulatory elements within the proximal 5' region of *xaf1 *promoter. Interestingly, under specific stress pressure, the heat-shock transcription factor 1 (HSF1) was demonstrated to function as a negative regulator of *xaf1 *expression in various gastroinstestinal cancer tissues and cell lines [28]. However, the potential influence of DNA methylation on HSF1 binding-mediated suppression of XAF1 transcription has not been studied. Conversely, XAF1 was found to be modulated positively (independently of DNA methylation) by all-trans retinoic acid (ATRA) and IFN through an IFN regulatory factor 1 binding element (IRF-E) that mediates transcription regulation and participates in ATRA-induced induced cell-growth suppression [29]. However a significant contribution of HSF1 or IRF-1 in IFN-β-mediated transient XAF1 expression observed in our study remains to be confirmed. On the other hand, IFN-β induces ISGs of which *xaf1 *is a member, through a JAK kinase cascade which in turn activates STAT factors [23,30]. Therefore, modifications in the activity of any of the members of the IFN-β cascade can be potentially responsible for modifications in *xaf1 *transcription that are independent of *xaf1 *promoter methylation.

We have demonstrated that endogenous XAF1 expression is crucial for the IFN-β-associated sensitization to TRAIL. The loss of XAF1 in three different shRNA stable glioblastoma cell lines completely abolished the sensitization effect of IFN-β observed in the parental SF539. The lack of XAF1 induction following IFN-β treatment in these stable cell lines demonstrates the efficacy of the shRNAs in suppressing XAF1 expression even in the presence of a proven inducer of the gene. Importantly, these findings allowed us to examine the relationship between endogenous XAF1, IFN-β and TRAIL-mediated apoptosis. Significantly, a previous report has demonstrated the critical role of XAF1 in overcoming resistance to IFN-β-induced apoptosis in the absence of DNMT1 expression, a result that supports our current findings [21].

Among the multitude of genes that are modulated by IFN [31,32] and can potentially enhance cell sensitivity to TRAIL-induced apoptosis, we show that *xaf1 *alone is important and essential in mediating susceptibility to TRAIL-induced apoptosis in cancer cells. Functional knockdown of *xaf1 *through shRNA is sufficient to eliminate the IFN-β-mediated sensitization to TRAIL in glioblastoma cells. Although TRAIL is a potent apoptotic trigger in many cancer cells, resistance to TRAIL, either intrinsic or acquired, currently represents a substantial limitation to therapy [33]. IFN treatment has been shown to overcome TRAIL resistance [34], however, resistance to the dual IFN/TRAIL therapy has also been observed [35]. In this context, the centrality of XAF1 in IFN-regulated apoptosis might be the key in reversing TRAIL-resistance.

## Conclusion

In conclusion, we demonstrate that, in spite of aberrant hypermethylation of the *xaf1 *gene, IFN-β was able to induce *xaf1 *expression. Furthermore, although promoter methylation is a good predictor to basal XAF1 expression, demethylation does not necessarily lead to sustained XAF1 expression. However, it is clear that XAF1 is an important mediator in sensitizing IFN-treated cells to TRAIL-induced killing. These results suggest that the incorporation of *xaf1 *in combination regimens might be of particular value in TRAIL-based anti-cancer strategies.

## Competing interests

The author(s) declare that they have no competing interests.

## Authors' contributions

OCM carried out the experiments and wrote the first draft. HHC did experiments and helped to draft the manuscript. SP provided technical expertise, helped to draft, and finalized the manuscript. SLH provided technical help at the DNA methylation analysis. EL aided in the generation of XAF1 shRNA vectors. PL and RGK both conceived and coordinated the study. All authors read and approved the final manuscript.

## Pre-publication history

The pre-publication history for this paper can be accessed here:
